# “What would you like to achieve?” Goal-Setting in Patients with Dementia in Geriatric Rehabilitation

**DOI:** 10.1186/s12877-019-1296-7

**Published:** 2019-10-22

**Authors:** Ilona Dutzi, Michael Schwenk, Marietta Kirchner, Jürgen M. Bauer, Klaus Hauer

**Affiliations:** 1 Department of Geriatric Research, AGAPLESION Bethanien Hospital, Geriatric Center at the University of Heidelberg, Rohrbacher Str. 149, 69126 Heidelberg, Germany; 2 Network Aging Research, University of Heidelberg, Bergheimer St. 20, 69115 Heidelberg, Germany; 3 Institute of Medical Biometry and Informatics, University of Heidelberg, Im Neuenheimer Feld 130.3, 69120 Heidelberg, Germany

**Keywords:** Dementia, Geriatrics, Rehabilitation, Goal-setting, ICF

## Abstract

**Background:**

Setting meaningful, individualized rehabilitation goals is an essential part of the rehabilitation process. Even though patients with dementia are a drastically increasing patient group in geriatric rehabilitation, empirical data about meaningful rehabilitation goals and collaborative goal-setting in this target group is missing. Cognitive impairment and lack of insight in current deficits have been discussed as barriers for participation in goal-setting, but require empirical examination.

This study investigated the feasibility of a semi-structured versus a structured goal-setting approach and the types of goals, rehabilitation patients with mild to moderate dementia perceive as personally relevant. Insights in acute functional and motor deficits, differentiated by cognitive status were explored.

**Methods:**

Cohort study in a geriatric rehabilitation center. Semi-structured and ICF-based, structured interviews were applied to explore patients` rehabilitation goals. Patients` insight in deficits was operationalized as the relationship of self-ratings and objective measures of linked clinical assessments for the same functional construct.

**Results:**

Patients (*n* = 101, MMSE 22 ± 2.6, age 83.9 ± 5.9 years) stated the improvement of mobility-related functions and self-care activities (> 70*%*) but also psychological well-being such as handling stress or mood (> 38*%)* as most important rehabilitation goals. The structured interview facilitated goal-setting and provided a broader view of rehabilitation needs. Correlations between self-ratings and clinical assessments were medium to high (*rho* = 0.29 to 0.83) with highest associations for key motor features. Trend tests identified a significant trend between values of the clinical assessment and categories of self-ratings (*p* ≤ 0.01) with lower cognitive status derogating this relationship.

**Conclusions:**

Collaborative goal-setting was feasible, especially when supported by a structured approach and yielded a large spectrum of functional but also psychological rehabilitation needs from the patients` perspective. Patients showed sustained insight in their actual functional impairments, limited in a subgroup of patients with more advanced cognitive impairment.

## Background

Geriatric rehabilitation (GR) specifically targets the rehabilitation of older persons after acute illness and hospitalization by means of multimodal and multiprofessional rehabilitation programs. Patients in geriatric post-acute rehabilitation are characterized by having complex care needs due to a high burden of comorbidities and pre-morbid functional limitations. The purpose is to protect or improve the patients’ health and quality of life, to optimize functional independence and to avoid admission to nursing home [1–3]. From a patient-centered perspective, successful GR aims to address those aspects of daily living considered most relevant by patients [4]. Therefore, the identification of goals in cooperation with the rehabilitant to individualize rehabilitation programs according to patients’ goals, values and resources is regarded as an essential part of the rehabilitation process [1, 2, 5, 6]. Among older inpatients, patients with dementia (PwD) are a drastically increasing sub-group in GR. Patients are characterized by immense individual differences in health problems and needs not only related to their main diagnosis at hand, but also due to their cognitive impairment, making an individualized approach particularly challenging.

Routinely used, formalized tools which guide and facilitate collaborative goal-setting in patients with dementia are missing. Instruments used in geriatric rehabilitation mostly are adopted from other clinical settings [7] and differ from one another regarding the professional group intended to use the approach, the process by which goals are selected, or the content of goals set. The most commonly used instruments in GR are the Goal Attainment Scale (GAS) [8] and the Canadian Occupational Performance Measure (COPM) [9]. But even if patient-centered goal identification is part of these tools, they have been primarily designed as an outcome measure in areas of planned interventions [10]. Accordingly, they show limited usefulness as a practice framework to guide clinicians through the process of collaborative goal-setting, strategies to support the identification and verbalization of potential rehabilitation goals were not specified.

In practice, the goal-setting process in geriatric rehabilitation still appears to be therapist-led rather than patient-based [5, 7, 11], leading to a systematic neglect of the patients´ perspective [12].

In the last years, numerous studies have been conducted to promote collaborative goal-setting and to gain insight in individual, meaningful goals for different patient-groups [13–19]. However, there is still a lack of data with regard to meaningful goals and the ability of PwD to actively participate in goal-setting in GR. Existing research with older patients often excluded PwD from study participation or subgroup analyses of patients with cognitive impairment are missing [5, 11, 12, 20–22]. Additionally, patient involvement in setting rehabilitation goals varies widely across studies. In most cases needs of patients were identified with discipline-specific assessments and goals were formulated during the team’s case conference [11, 20, 21]. One of the few studies that directly involved geriatric rehab patients in goal-setting used a semi-structured interview with open ended questions [22]. Patients’ goal statements were linked to the International Classification of Functioning, Disability and Health (ICF) [23] and clustered into different goal categories. In this study “mobility related activities”, “getting rid of pain”, “autonomy” and “returning home” were the most frequently reported goals by patients. However, it remained unclear whether results were generalizable to PwD for different reasons. First, even if patients with dementia were not systematically excluded, the study population was a positive selection towards mentally fit persons and subgroup analyses for patients with cognitive impairment were not conducted. Second, as cognitive and communication deficits were identified as personal barriers to articulate needs and involvement with goal-setting [24–27], it might be questioned whether patients with deficits in memory, language and executive functions typical for dementia, are able to participate in such a cognitive demanding semi-structured approach. Tailoring the process to patient’s cognitive deficits by using communication strategies or the provision of a structured interview material was found to be helpful in patients with stroke [25] and might equally facilitate the identification of rehabilitation goals in PwD but needs empirical examination. The same is true for another commonly suggested barrier of collaborative goal-setting in PwD, namely the lack of patients` insight into acute deficits [25, 27]. The ability to understand functional problems, the rehabilitation process and the potential for recovery generally is regarded as an ability typically deteriorating in the course of dementia [27]. But while a considerable amount of studies analyzing awareness notably for cognitive and memory dysfunction and found a high percentage of patients who overestimated their abilities [28–31], research on the perception of everyday functional abilities is scarce and provided more heterogeneous results. Some authors reported only modest correlation between self-reports and actual performance in everyday competence [32] and an overestimation of abilities in self-report, increasing with the severity of dementia [33], whereas others revealed moderate to high correlations between self-ratings and objectively assessed instrumental activities of daily living [34]. However, to the best of our knowledge, there are no studies in the rehabilitation setting examining patients` insight in acute functional and motor impairments of PwD as a mandatory step for goal-setting during the rehabilitation process.

Although doubts are plausible, the ability of elderly patients with dementia in geriatric inpatient rehabilitation, to participate in collaborative goal-setting, is still a largely unanswered empirical question.

Based on the identified knowledge gaps with regard to rehabilitation goals and collaborative goal-setting in PwD the aims of the current study were (I) to investigate the feasibility of a semi-structured vs. structured goal-setting approach in patients with mild to moderate dementia in geriatric rehabilitation and to identify types of goals patients perceive as personally relevant; (II) to explore patients` insight in acute functional and motor deficits, operationalized as the relationship of self-ratings of functional abilities and measures of objective clinical assessments of the same construct; and (III) to investigate if this relationship is influenced by cognitive status.

## Methods

### Study design and setting

We present a prospective cohort study of geriatric inpatients with dementia, consecutively recruited during ward-based rehabilitation between 04/2011 and 12/2011 with data collection starting within 48 h after admission. The study was part of the model project “Geriatric Rehabilitation for Demented Patients Study” (GREDE) conducted at the AGAPLESION Bethanien Hospital / Geriatric Center, University of Heidelberg, Germany [35, 36].

The inclusion criterion for GREDE was the diagnosis of mild to moderate dementia as a secondary diagnosis according to core criteria for all-cause dementia [37].

Exclusion criteria were medical and/or psychological conditions not allowing neuropsychological and motor-functional assessments, such as acute confusion (delirium), aphasia, severe visual or auditory impairment, severe psychiatric disorders, inadequate language level, severe functional-motor deficits, lower limb fractures with partial weight bearing or severe acute medical conditions and no written consent to participate in the study.

Positive screened persons and, if applicable, their existing legal guardians or authorized representatives were informed in verbal and written form about the study program and asked to give written consent before inclusion. The whole procedure was conducted in a comprehensible way according to the recommendations of Appelbaum [38].

### Measures and data collection procedures

#### Patient characteristics

Patient characteristics included admission age, gender, number of medications, indication for geriatric rehabilitation by diagnostic groups as documented in patient charts, depressive symptoms (Geriatric Depression Scale, 15-item version [39]), frailty status (Clinical Frailty Scale, range 1–9 [40]), length of stay (number of days), living arrangement (community dwelling vs. institutionalized). The Mini-Mental State Examination (MMSE) was used as a screening instrument to assess participants’ cognitive function (range 0 to 30, higher scores indicating better cognitive function [41]). Activities of daily living were evaluated by the Barthel Index administered by observation of the patients` performance by a trained nurse (maximal score 100) [42, 43].

#### Assessment of relevant goals and self-rating of functional abilities from the patients` perspective

Relevant goals from the patient’s perspective were assessed in two ways: First, comparable to the aforementioned study of Kus et al. [22], we conducted a semi-structured interview with open-ended questions. Second, to facilitate the identification and verbalization of potential rehabilitation goals, we applied a tailored goal-setting approach using a structured interview with pre-prepared questions and examples of potential rehabilitation goals.

Additionally, patients were asked to rate severity of perceived problems for each goal area.

##### Semi-structured goal-assessment

Participants were encouraged to report important and individually relevant rehabilitation goals related to their health condition and hospitalization. They were not limited as to the number of statements. To assess the extent to which patients were able to provide rehabilitation goals, the number of individual goal statements was documented. Each interview began with the question: “What are your goals for your rehabilitation?” If participants appeared to have difficulty understanding the term “goal,” alternative terms were offered, such as “desired personally relevant outcomes” or “what would you like to achieve during rehabilitation.” For data analysis, patients’ statements were translated into the ICF terminology and linked to the most closely corresponding ICF categories and respective codes following a standardized linking procedure [44, 45]. In cases where a patient’s goal could not be attributed to the ICF, e.g. because the statement was too general for linkage, or if the content was not covered by the ICF, we summarized and grouped this data as “not definable”.

##### Structured goal-assessment

As the ICF was found to be a helpful tool to identify and structure health problems from a patient’s perspective in other studies in the rehabilitation setting [22, 46–48], we applied an ICF-oriented framework for the structured goal assessment, too. For description and assessment of patients` problems, there exists a comprehensive ICF Core Set, which includes 123 categories, relevant for functioning in patients in geriatric post-acute rehabilitation facilities [49, 50]. For feasibility in clinical practice, the application of a shorter subset, selected according to the specific needs of individual users is recommended [51]**.** To gain a holistic view of rehabilitation needs, we selected 19 categories covering areas of “motor functioning” (6 items), “self-care” (5 items), “domestic life” (1 item), “psychological well-being” (3 items), “sensory functions” (1 item), “social relationship” (2 items) and “cognition” (1 item) as potential rehabilitation goals in a consensus process by three health care professionals familiar with the ICF Core Set and the rehabilitation of patients with dementia. Items and respective ICF chapters and categories used in the structured questionnaire are shown in Table 1. Patients were asked to weight the goal categories with regard to the relevance for their daily life (rated from (0) not relevant to (1) relevant and (2) very relevant as rehabilitation goal). Each item followed the same pre-prepared question: “How relevant is it for you to improve your ... (e.g. item 4: walking ability?)”

**Table 1:** Items and categories used in the structured ICF-oriented questionnaire

Item	Content	ICF chapters and categories	ICF Codes
1. Mobility	Motor functions	Mobility; Changing and maintaining body positions	d410 d415
2. Lower extremity muscle power function	Motor functions	Neuro-musculoskeletal and movement-related functions; using the legs and feet to exert a force on an object to move it away; getting into and out of a seated position	b7303 d435 d410
3. Fine hand use	Motor functions	Mobility; Grasp small objects with fingers and hands and pick up small objects (for example coins), button clothes	d440
4. Walking	Motor functions	Mobility; walking short or long distances without walking aids	d450 d460
5. Walking around with walking aid	Motor functions	Mobility; walking short or long distances by using specific devices like walker or walking sticks	d465
6. Personal hygiene	Self-care	Self-care; Washing oneself Caring for body parts; Toileting	d510 d520 d530
7. Dressing	Self-care	Self-care; putting on or taking off clothes and footwear	d540
8. Eating and drinking	Self-care	Self-care; Eating and Drinking without any help and without any difficulty	d550 d560
9. Household tasks	Domestic life	Domestic life; preparing simple meals and doing housework; wash dishes, cleaning cooking area and utensils;	d6300 d6340
10. Physical endurance	Motor functions	Functions of the cardiovascular, haematological, immunological and respiratory systems; functions of physical endurance, aerobic capacity, stamina and fatigability	b455
11. Pain	Sensory functions	Sensory functions and pain; sensations of generalized or localized pain in one or more body part,	b280
12. Mood/ Depressive symptoms	Psychological wellbeing	Mental functions; regulation and range of sadness, lability of emotion	b152
13. Anxiety, fear of falling	Psychological wellbeing	Mental functions; functions of appropriateness of anxiety, fear of falling	b152
14. Handling stress and psychological demands	Psychological wellbeing	General tasks and demands; Handling stress and psychological demands	d240
15. Memory and attention	Cognition	Mental functions; concentration; sustaining and shifting attention Registering, storing and retrieving information; short- and long-term memory; remembering;	b140 b144
16. Family relations	Social relationship	Interpersonal interactions and relationships; parent-child and child-parent relationships, sibling and extended family relationships	d760
17. Informal social relationships	Social relationship	Interpersonal interactions and relationships; entering into relationships with others, such as casual relationships with people living in the same community or residence, friends, neighbours	d750
18. Knowledge about acute and chronic illnesses	Self-care	Self-care; maintaining one’s health	d5702
19. Knowledge about medication, assistive products	Self-care	Self-care; maintaining one’s health	d5702

The ICF has two parts, each containing two separate components. Part 1 covers functioning and disability and includes the components Body Functions (b), Body Structures (s) and Activities and Participation (d). Part 2 covers contextual factors and includes the components Environmental Factors and Personal Factors (e). In the ICF classification, the letters b, s, d, and e, which refer to the components of the classification are followed by a numeric code that starts with the chapter number (one digit), followed by the second level (two digits), as well as third and fourth levels (one extra digit each). Digits in column 4 refer to components of the ICF followed by numeric codes

##### Self-rating of functional abilities

In addition, all patients were asked for health evaluations in each category using ICF qualifiers on a five-point rating scale. ICF qualifiers denote the magnitude of the level of health or severity of a problem and were coded as (0) no, to (1) mild, (2) moderate, (3) severe problems or (4) complete problem [52].

Before the actual start of the study, interviewers were trained in principles of the ICF, communication strategies and interview technique to ensure an effective and standardized procedure. Role playing games and practice phase with voluntary test persons were conducted. During the training sessions, personal instruction and feedback were given to the interviewers. Ongoing supervision of the interviewers was granted. As the aim of the study was to strictly focus on the patient’s perspective, all interviews were conducted without the presence of a family member, proxy or care giver.

### Objective measures of functional abilities

To explore patients` insight in functional deficits we identified clinical assessment instruments matching the items of motor function and self-care according to established linking rules and literature reviews [44, 45, 48, 52, 53]. Deficits in “fine hand use” was not assessed by clinical measurements for all patients and could therefore not be taken into account.

The Hierarchical Assessment of Balance and Mobility (HABAM) (maximal score 67) [54] and Tinetti’s Performance Oriented Motor Assessment (POMA) [55] Balance sub-scale (maximal score 16) were used to assess motor features such as balance and transfer and were linked to item 1 “Mobility”. Both assessments are common clinical tests for assessing mobility deficits in older persons by external expert ratings with higher scores indicating less functional deficits. “Lower extremity strength” was linked to a standardized One Repetition Maximum (1RM) achieved at a leg-press training machine for maximal dynamic strength in hip and knee extensors (in kg) (Kaphingst, Lahntal, Germany) and the 5 chair-stand (time needed to rise from a chair and sit down 5 times consecutively measured in seconds) [53, 56]. “Walking ability” was objectified with two performance-based measures: gait speed (cm/sec) was assessed with the electronic GAITRite-analysis system (CIRSystems, Havertown, PA; length: 4.8 m), where subjects had to perform two walks with maximum walking speed [57] and the Timed up and Go test (TuG) which measures time taken (in seconds) to stand up from a regular arm chair, walk a 3-m distance at a comfortable pace, turn around, return to the chair and sit down again [53, 58]. Self-care was operationalized by three sub-items of the Barthel Index: “Feeding” and “Dressing” (unable (0), needs some help (5), independent (10)), and “Personal Hygiene” (unable (0), independent (5)).

### Data analysis

Sociodemographic and clinical baseline characteristics of participants were documented as frequencies (*n, %*), means with standard deviations or medians with interquartile ranges as appropriate.

To evaluate patients` insight in acute functional deficits, the relationship of self-ratings and objective measures were analyzed. Summary statistics of the subjective and objective measures were provided which, in case of the objective measures, were additionally stratified by the five categories of the self-rating. To investigate, if there was a statistically significant trend between values of the objective measures and subjective evaluations, the Jonckheere trend test (JT) was applied and the respective two-sided *p*-values were reported. The JT is a rank-based, nonparametric test for an a priori ordered alternative hypothesis within a between-participants design and was used to determine if scores in objective assessments decreased according to increasing subjectively perceived functional problems. Spearman’s correlation coefficient *rho* was calculated as a measure of the strength of the respective relationship. Coefficients were interpreted as low (*rho* < 0.2), moderate (*rho* = 0.2–0.5), or high (*rho* > 0.5) [59].

To evaluate whether the relationship of self-ratings and objective measure was influenced by cognitive status of participants, we divided the sample using the median split of MMSE as a sample specific cut-off and conducted separate bivariate correlations and trend tests for participants with lower (≤ median) and higher (> median) cognitive status.

Analyses were based on the complete-case data set; missing values were not imputed. This is an exploratory analysis; all *p*-values are interpreted descriptively and a two-sided *p*-value ≤0.05 indicates statistical significance Statistical analysis was performed using the SPSS statistics 25.0 (IBM, Armonk, NY, USA).

## Results

### Participants

The study included 107 multi-morbid, GR patients (age 83.9 ± 5.9 years) with mild to moderate dementia (MMSE 21.9 ± 2.6) and impaired functional status (ADL-score 58.3 ± 19.0), typical for geriatric rehabilitation. Due to cognitive limitations *n* = 6 (5*%)* patients were unable to cope with the interviews. These patients had problems to come to a real understanding of the questions or were confused by questions so that answers were not evaluable. Patients who dropped out showed more advanced cognitive impairments in comparison to patients who could participate in interviews (MMSE 19.2 ± 0.8 versus 22.0 ± 2.6, *p* = 0.01). Demographics and clinical characteristics of the study population are presented in Table 2.

**Table 2:** Sociodemographic and clinical baseline characteristics of the study population

Characteristic	Value
Age, years, mean ± *SD*	83.9 ± 5.9
Gender, female, *%*	81.1
Mini-Mental State Examination (MMSE) Score (0–30), median *(IQR)*	22 (20–24)
Living arrangement before admission, *n, %*	
Institutionalized	12 (11.3)
Community dwelling	94 (88.7)
Number of medications, *n*, mean ± *SD*	9.4 ± 3.2
Clinical frailty scale, Score (1–9), median (*IQR*)	6 (5–6)
Admission main diagnosis, *n*, *%*	
Orthopedic impairment	41 (38)
Cerebrovascular disease	22 (20)
Heart disease	17 (16)
Other internal disease	13 (12)
Miscellaneous	14 (13)
Geriatric Depression Scale (GDS) Score (0–15), median (*IQ*R)	4 (2–5)
Barthel Activity of Daily Living (ADL) Score (0–100), median (*IQR*)	60 (44–70)
Length of stay during geriatric rehab, days, median *(IQR)*	20 (19–26)

Note. Higher scores indicating less impairment in MMSE and ADL; for frailty and GDS higher scores indicate higher degree of symptoms; SD, Standard Deviation; IQR, Interquartile range

### Assessment of relevant goals

#### Semi-structured goal-assessment

The remaining 101 participants provided 143 goal statements in total. 18 patients did not provide any goal, 34 provided one goal each, 39 two, 9 three and 1 patient provided four goals (mean = 1.4; median = 1). Patients who did not provide any statement did not differ significantly from those who provided at least one goal with regard to their cognitive status (MMSE scores 21.3 ± 2.4 vs. 22.2 ± 2.6; *p* = .19). The majority of goals (78*%)* could be linked to ICF-categories (see Table 3). Goals covered the ICF domains mobility, mental functions, sensory functions and pain, functions of the cardiovascular system, neuro-musculoskeletal and movement-related functions, self-care, domestic life, relationships, general tasks and demands, communication, and support from the ICF components activities and participation, body functions and structures and environmental factors. Most statements and respective categories appeared in the mobility- (50*%*) and pain domain (10*%*). Statements ranged from very general goals (“improve walking ability”) to detailed descriptions of specific aspects of functioning (“to climb the 80 steps to my house”). Other categories were named with a frequency of less than 5*%*. Several goal statements (22*%*) were not linkable to ICF categories but could be summarized as a general longing for autonomy and optimization of their health condition without further specifications.

**Table 3:** Patient goals assessed by semi-structured goal assessment linked to International Classification of Functioning, Disability and Health (ICF) categories

Goals grouped by ICF chapters	*n (%)*	ICF codes and categories
Mobility	72 (50)	
Mobility and Balance	12	d410, 415 changing and maintaining body positions, d420 transferring oneself
Moving around	21	d460, 465 Moving around in different locations
Walking	31	d450 Walking, d4502 Walking on different surfaces d4501 Walking long distances
Stair climbing (80 steps)	2	d4551 Climbing
Driving a car	2	d475 Driving
Mobility of hand and arm	2	d445 Hand and arm use
Swimming	2	d4554 Swimming
Domestic life	6 (4)	
Gardening	1	d6505 Taking care of plants outdoors
To do the housework	4	d630 Preparing meals, d640 Doing housework
Sewing	1	d6500 Making and repairing clothes
Self-care	5 (3)	
Toileting	3	d530 Toileting
Bathing, showering	1	d510 Washing oneself
Putting on or taking off clothes	1	d540 Dressing
Relationship	2 (1)	
To socialize	2	d750, d760 Informal and family relationship
General tasks and demands	2 (1)	
To recharge one’s battery to carry out and manage everyday tasks	2	d240 handling stress
Communication	1 (< 1)	
Communicate with others by language	1	d330 speaking
Mental functions	2 (1)	
Regulation of depressive symptoms	1	b152 Emotional functions
Memory, cognition	1	b144 Memory functions
Sensory function and pain	13 (9)	
Sensation of generalized or localized pain	7	b280 Pain
Visual functions	1	b210 Seeing functions
Dizziness	5	b240 Dizziness and vertigo
Functions of the cardiovascular system	4 (3)	
Physical endurance, fatigability	4	b455 Exercise tolerance functions
Neuromusculoskeletal and movement related functions	2 (1)	
Handgrip	2	b730 Muscle power functions
Support and relationship	2 (1)	
Organize support and care for the post-hospitalization phase	2	e340 Personal care providers and personal assistants
Goals unrelated to ICF coding	32 (22)	
General health/ convalescence	10	Not definable
Autonomy/ returning home	22	Not definable

Given are absolute frequencies of ICF categories linked to patient goals (*n* = 143). Relative frequencies are given for ICF chapters. Characters in column 1 and 3 refer to components of the ICF, where the code appears: b = Body Functions (physiological functions of body systems); d = Activities and Participation (execution of tasks or actions and involvement in a life situation); e = Environmental and Personal Factors (physical, social and attitudinal environment). Numeric codes start with the chapter number (first level category, one digit), followed by the second level (two digits), as well as third (one extra digit)

#### Structured goal-assessment

**Fig. 1:**
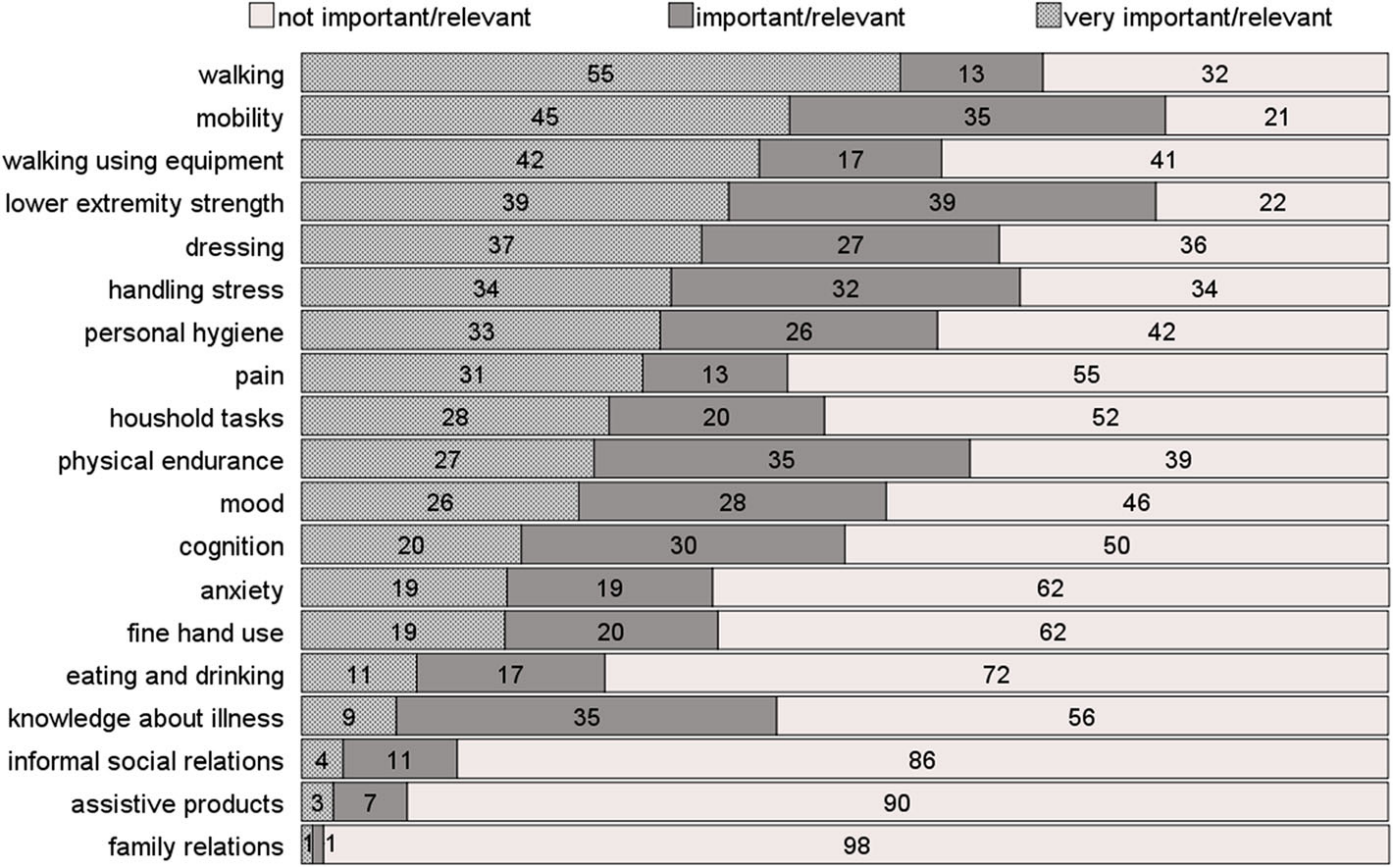
Valuation of relevance of ICF-oriented goal areas. Given are frequencies (in *%*) of valuation for each category

Results of the structured goal-assessment by means of a tailored structured interview with pre-prepared questions and examples of ICF-based potential rehabilitation goals gave a broader view of patients` rehabilitation needs. Figure 1 illustrates patients` weighting of selected goal areas, arranged in order of perceived personal relevance. Items covering motor and mobility-related functions (walking, changing and maintaining body positions, lower extremity strength) were rated as relevant by almost 70*%* of patients. Additionally, items related to psychological wellbeing (mood, handling stress), cognition (memory and attention) and self-care (taking on and off clothes, personal hygiene, and household tasks) were rated as relevant rehabilitation goals by more than 50*%* of patients. Informal and family relationships as well as knowledge on assistive products were perceived as least relevant categories (< 10*%*).

#### Self-ratings of functional abilities

Most patients reported problems in the motor items “walking” (95*%*), “lower extremity muscle strength” (88*%*), “mobility” (88*%*) and specific items of self-care as “household tasks” (89*%*)*,* “taking on and off clothes” (74*%*) and “personal hygiene” (72*%*)*.* But also in cognitive (73*%*) and the psychological functions “handling stress” (73*%)* and “mood” (68*%*), a high percentage of patients indicated health problems. Problems were perceived with lowest frequency in informal (27*%*) and family relationships (14*%*) (see Table 4).

**Table 4:** Self-rating of functional abilities and deficits

Goal area/Item	Category of self-rating *(%)*					Median (Min-Max)
	0	1	2	3	4	
Walking	4.0	15.0	7.0	20.0	54.0	4 (0–4)
Household tasks	10.6	18.8	12.9	17.6	40.0	3 (0–4)
Mobility	11.9	29.7	18.8	30.7	8.9	2 (0–4)
Lower extremity strength	11.9	26.9	30.7	23.8	6.9	2 (0–4)
Taking on and off clothes	25.5	22.4	24.5	15.3	12.2	2 (0–4)
Walking around with equipment	37.8	23.50	17.3	12.2	9.2	1 (0–4)
Physical endurance	19.8	30.2	29.2	15.6	5.2	1 (0–4)
Handling stress and psychological demands	26.9	28.9	27.8	11.8	4.3	1 (0–4)
Pain	43.9	20.4	13.3	19.4	3.1	1 (0–4)
Personal hygiene	28.0	32.0	21.0	16.0	3.0	1 (0–4)
Knowledge about acute and chronic illnesses	46.2	26.9	21.5	4.3	1.1	1 (0–4)
Fine hand use	48.9	28.7	17.0	4.3	1.1	1 (0–4)
Mood/ Depressive symptoms	32.0	42.3	12.4	12.4	1.0	1 (0–4)
Memory and attention	26.8	45.4	16.5	11.3	0.0	1 (0–3)
Knowledge about medication, assistive products	52.4	18.3	15.9	7.3	6.1	0 (0–4)
Anxiety, fear of falling	52.0	26.5	11.2	8.2	2.0	0 (0–4)
Eating and drinking	63.6	23.2	5.1	6.1	2.0	0 (0–4)
Informal social relationships	77.1	17.7	4.2	1.0	0.0	0 (0–3)
Family relations	85.9	6.5	4.3	3.3	0.0	0 (0–3)

Note. Given are relative frequencies of reported problems assessed with the ICF oriented structured interview: 0 = no, 1 = mild, 2 = moderate, 3 = severe, 4 = complete problem; Items are hierarchically arranged by perceived impact (Median)

#### Relationship between self-ratings and objective measures

Bivariate correlations revealed significant moderate to high associations between all subjective health evaluations and objective clinical measures of the same functional construct, indicating a considerable insight in functioning and deficits in the target group: More severe subjective health problems were associated with worse results in the respective clinical assessment. Highest associations were found for self-ratings of key motor functions such as “walking problems” with GaitRite speed (*rho* = −0.83) and the Timed up and go test respectively (*rho* = 0.73). Jonckheere tests specified these results by the determination of highly significant trends between categories of self-ratings and the linked clinical measures (*p* ≤ 0.001 to 0.005) indicating that objective measures differed according to categories of self-ratings (see Table 5).

**Table 5:** Summary statistics for objective clinical measures stratified by values of self-rating

Clinical Measure	All	Category of self-rating					JT *p*-value	*rho*
		0	1	2	3	4		
MOTOR FUNCTIONS								
HABAM, score								
*n*	100	11	30	19	31	9	< 0.001	−0.43**
Median	38	46	49.5	33	33	29		
*IQR*	29–50	35–58	36–53	28–47	26–42	21–29		
POMA Balance, score								
*n*	99	11	30	18	31	9	< 0.001	−0.46**
Median	11	14	13	10	7	5		
*IQR*	6–14	11–15	11–14	5–13	4–12	2–7		
LOWER EXTERMITY MUSCLE STRENGTH								
5 chair rise, s								
*n*	61	11	21	16	10	3	0.001	0.41**
Median	15.6	11.5	15.6	17.0	17.6	15.9		
*IQR*	12.6–20.4	10.7–12.6	13.5–18.2	14.3–22.3	14.7–22.2	12.9–26.7		
1-RM, kg								
*n*	90	12	24	27	21	6	0.005	−0.30**
Median	80	135	80	70	70	50		
*IQR*	60–130	85–190	65–105	50–130	60–110	40–70		
WALKING								
TuG, s								
*n*	78	4	15	7	20	31	< 0.001	0.73**
Median	21.9	8.2	15.9	17.7	21.3	34.8		
*IQR*	16.1–30.8	7.8–10.6	11.2–19.6	14.0–23.8	16.8–25.4	26.0–41.9		
GaitRite, speed cm/s								
*n*	86	4	15	7	20	39	< 0.001	−0.83**
Median	64.3	171.3	88.2	73.2	65.3	47.3		
*IQR*	45.0–84.8	139.2–176.2	75.6–135.4	60.1–96.9	57.9–78.4	27.4–65.8		
PERSONAL HYGIENE								
BI “Personal Hygiene”, score							0.005	−0.29**
*n*	99	27	32	21	16	3		
0, *n (%)*	17 (17.2)	0 (.)	7 (21.9)	3 (14.3)	6 (37.5)	1 (33.3)		
5, *n (%)*	82 (82.8)	27 (100)	25 (78.1)	18 (85.7)	10 (62.5)	2 (66.7)		
TAKING ON AND OFF CLOTHES								
BI “Dressing”, score								
*n*	98	25	22	24	15	12	< 0.001	−0.44**
0, *n* (*%*)	21 (21.4)	2 (8)	4 (18.2)	4 (16.7)	5 (33.3)	6 (50)		
5, *n* (*%*)	55 (56.1)	11 (44)	12 (54.5)	16 (66.7)	10 (66.7)	6 (50)		
10, *n* (*%*)	22 (22.4)	12 (48)	6 (27.3)	4 (6.7)	0 (.)	0 (.)		
EATING AND DRINKING								
BI “Feeding”, score								
*n*	98	62	23	5	6	2	0.002	−0.31**
0, *n* (*%*)	1 (1)	0 (.)	0 (.)	0 (.)	1 (16.7)	0 (.)		
5, *n* (*%*)	39 (39.8)	19 (30.6)	12 (52.2)	2 (40)	4 (66.7)	2 (100)		
10, *n* (*%*)	58 (59.2)	43 (69.4)	11 (47.8)	3 (60)	1 (16.7)	0 (.)		

Given are *p*-values for the Jonckheere test (JT) for significant ordered values and Spearman’s correlation coefficient *rho* for bivariate correlations between values of the objective measures and subjective valuations for the whole sample ** = *p* < 0.01for correlations; Categories of self-ratings: 0 = no, 1 = mild, 2 = moderate, 3 = severe, 4 = complete problem; IQR, Interquartile range; 1 RM, leg press, HABAM TS, Hierarchical Assessment of Balance and Mobility (range 0–67); POMA, Tinetti’s Performance Oriented Motor Assessment, Sub-score Balance (range 0–16); TuG, Timed up and go test; BI, Barthel Index sub-items; feasibility of motor testing was partly limited due to motor limitations, medical reasons, or lack of motivation which reduced the sample size for specific outcomes

#### Influence of cognitive status

In an analysis of two subpopulations, dichotomized for cognitive status (MMSE ≤22 vs. > 22), correlations between subjective and objective measures as well as trend tests revealed somewhat weaker but still significant associations between self-ratings and objective measurements in both subsets for nearly all categories, indicating a sustained insight in functional deficits in patients with mild to moderate dementia. However, for “lower extremity muscle strength” and self-care “eating and drinking” and “personal hygiene” associations and trends in patients with lower MMSE scores did not reach statistical significance suggesting partly reduced insight of patients with lower cognitive status (see Table 6).

**Table 6:** Summary statistic for objective clinical measures stratified by values of self-rating and cognitive status with

Clinical measure		All	Category of self-rating					JT *p*-value	*rho*
			0	1	2	3	4		
MOTOR FUNCTIONS									
HABAM, score									
MMSE 17–22	*n*	53	6	17	6	19	5	0.002	−0.44**
	Median	33	40.5	50	28.5	33	21		
	*IQR*	28–50	35–50	39–53	28–33	26–46	17–29		
MMSE 23–26	*n*	47	5	13	13	12	4	0.004	−0.42**
	Median	40.5	50	47	33	32.5	41		
	*IQR*	29–53	46–58	36–53	32–47	24.5–40	25–53		
POMA Balance, score								0.002	−0.43**
MMSE 17–22	*n*	52	6	17	5	19	5		
	Median	10	12	13	5	8	5		
	*IQR*	5–13.5	7–14	12–15	4–8	2–12	2–6		
MMSE 23–26	*n*	47	5	13	13	12	4	0.001	−0.47**
	Median	11	14	12	11	6	9		
	*IQR*	6–14	14–15	11–14	7–14	4.5–11	2.5–14		
LOWER EXTERMITY MUSCLE STRENGTH									
5 chair rise, s									
MMSE 17–22	*n*	29	5	13	8	2	1	0.155	0.26
	Median	14.1	12.6	15.2	15.1	15.5	26.7		
	*IQR*	11.8–19.1	10.7–14.1	13.1–18.0	11.6–21.3	11.8–19.1	26.7		
MMSE 23–26	*n*	32	6	8	8	8	2	0.021	0.42*
	Median	16.0	11.4	16.3	17.5	18.4	14.4		
	*IQR*	13.8–20.6	11.0–11.8	15.5–19.4	16.3–25.9	14.9–24.1	12.9–15.9		
1-RM, kg									
MMSE 17–22	*n*	48	6	16	16	8	2	0.295	−0.16
	Median	90	135	80	80	80	90		
	*IQR*	60–135	90–180	60–105	60–125	60–130	60–120		
MMSE 23–26	*n*	42	6	8	11	13	4	0.009	−0.40**
	Median	70	145	80	60	70	40		
	*IQR*	50–120	80–210	70–105	30–130	50–110	30–55		
WALKING									
TuG, s								< 0.001	0.84**
MMSE 17–22	*n*	38	1	11	3	7	16		
	Median	21.9	7.9	13.4	14.0	23.7	35.0		
	*IQR*	14.0–31.3	7.9	11.2–18.3	12.4–15.4	21.4–26.3	27.5–40.1		
MMSE 23–26	*n*	40	3	4	4	13	15	< 0.001	0.58**
	Median	22.0	8.6	19.0	21.9	20.9	34.7		
	*IQR*	17.6–30.3	7.8–12.7	14.5–30.8	18.9–26.8	16.1–22.6	23.2–45.2		
GaitRite, speed, cm/s									
MMSE 17–22	*n*	42	1	11	3	7	20	< 0.001	−0.70**
	Median	67.7	170.2	93.8	96.9	60.3	48.9		
	*IQR*	47.3–89.7	170.2	80.5–135.5	85.0–103.6	50.4–89.7	31.6–62.5		
MMSE 23–26	*n*	44	3	4	4	13	19	< 0.001	−0.54**
	Median	64.3	172.3	77.9	62.5	66.2	44.8		
	*IQR*	44.9–76.7	108.1–180.1	62.4–107.8	56.8–69.0	62.5–77.6	27.4–67.3		
PERSONAL HYGIENE									
BI “Personal Hygiene”, score									
MMSE 17–22	*n*	52	12	21	10	7	2	0.126	−0.22
	0, *n* (*%*)	9 (17.3)	0 (.)	5 (23.8)	1 (10)	2 (28.6)	1 (50)		
	5, *n* (*%*)	43 (82.7)	12 (100)	16 (76.2)	9 (90)	5 (71.4)	1 (50)		
MMSE 23–26	*n*	47	15	11	11	9	1	0.016	-0,35*
	0, *n* (*%*)	8 (17.0)	0 (.)	2 (18.2)	2 (18.2)	4 (44.4)	0 (.)		
	5, *n* (*%*)	39 (83.0)	15 (100)	9 (81.8)	9 (81.8)	5 (55.6)	1 (100)		
TAKING ON AND OFF CLOTHES									
BI “Dressing”, score									
MMSE 17–22	*n*	51	13	13	12	6	7	0.031	−0.30*
	0, *n* (*%*)	12 (23.5)	2 (15.4)	3 (23.1)	2 (16.7)	2 (33.3)	3 (42.9)		
	5, *n* (*%*)	32 (62.7)	7 (53.8)	8 (61.5)	9 (75.0)	4 (66.7)	4 (57.1)		
	10, *n* (*%*)	7 (13.7)	4 (30.8)	2 (15.4)	1 (8.3)	0 (.)	0 (.)		
MMSE 23–26	*n*	47	12	9	12	9	5	< 0.001	−0.60**
	0, *n* (*%*)	9 (19.1)	0 (.)	1 (11.1)	2 (16.7)	3 (33.3)	3 (60)		
	5, *n* (*%*)	23 (48.9)	4 (33.3)	4 (44.4)	7 (58.3)	6 (66.7)	2 (40)		
	10, *n* (*%*)	15 (31.9)	8 (66.7)	4 (44.4)	3 (25)	0 (.)	0 (.)		
EATING AND DRINKING									
BI “Feeding”, score									
MMSE 17–22	*n*	52	30	16	2	4	0	0.062	−0.26
	0, *n* (*%*)	1 (1.9)	0 (.)	0 (.)	0 (.)	1 (25)	0 (.)		
	5, *n* (*%*)	23 (44.2)	11 (36.7)	9 (56.3)	1 (50)	2 (50)	0 (.)		
	10, *n* (*%*)	28 (53.8)	19 (63.3)	7 (43.8)	1 (50)	1 (25)	0 (.)		
MMSE 23–26	*n*	46	32	7	3	2	2	0.018	−0.35*
	0, *n* (*%*)	0 (.)	0 (.)	0 (.)	0 (.)	0 (.)	0 (.)		
	5, *n* (*%*)	16 (34.8)	8 (25)	3 (42.9)	1 (33.3)	2 (100)	2 (100)		
	10, *n* (*%*)	30 (65.2)	24 (75)	4 (57.1)	2 (66.7)	0 (.)	0 (.)		

Give are *p*-values for the Jonckheere test (JT) for significant ordered values and Spearman’s correlation coefficient *rho* for bivariate correlations between values of the objective measures and subjective valuations ** = *p* < 0.01, * = *p* < 0.05 for correlations; Categories of self-ratings: 0 = no, 1 = mild, 2 = moderate, 3 = severe, 4 = complete problem; MMSE, Mini-Mental State Examination; IQR, Interquartile range; 1 RM, leg press; HABAM, Hierarchical Assessment of Balance and Mobility (range 0–67); POMA, Tinetti’s Performance Oriented Motor Assessment, Sub-score Balance (range 0–16); TuG, Timed up and go test; BI, Barthel Index sub-items. Feasibility of motor testing was partly limited due to motor limitations, medical reasons, or lack of motivation which reduced the sample size for specific outcomes

## Discussion

To the best of our knowledge, this is the first study investigating goal-setting in geriatric rehabilitation inpatients with dementia. Our main results can be summarized as follows: (I) goal-setting in patients with mild to moderate dementia was feasible, especially when patients were supported by a structured approach, which yielded a more holistic view of potential rehabilitation goals and needs from the patients` perspective. Mobility related functions were stated as the most important rehabilitation goals, followed by functions related to psychological well-being. (II) Self-reported functional problems showed a significant relationship with objective clinical assessments indicating a sustained insight in acute functional deficits in patients with mild to moderate dementia. (III) The strength of this relationship was partly reduced in the subgroup of patients with more advanced cognitive impairment.

### Assessment of relevant goals

In the semi-structured interview participants stated improvements in activities associated with mobility, especially walking and moving around, the reduction of pain, autonomy, and improving their general health condition as their most important goals. This finding confirmed results of previous studies conducted with geriatric inpatients without defined cognitive status [22]. However, with the unstructured approach 18*%* of patients did not provide any goal at all and one third of patients provided only one statement. The mean rate of one statement per patient was strikingly low, when compared to studies using the same method in mentally fit patients, with less than 10*%* of patients with no goal statement and a mean of 3 goals per patient [22, 46].

In comparison to the unstructured approach, the application of a structured interview, using pre-prepared questions and selected categories of the ICF as examples of potentially relevant rehabilitation goals resulted in a more holistic view of rehabilitation needs from the patients` perspective and was feasible for all patients. The discrepancy between the two approaches suggested that the tailored approach facilitated the identification of relevant rehabilitation goals in our patient group and strongly supports the need of alternative, tailored strategies for goal-setting in patients with mild to moderate cognitive impairments. It conforms with and expands the finding of a systematic review and meta-synthesis on barriers and facilitators to goal-setting during stroke rehabilitation that the use of structured methods and supporting material can facilitate the goal-setting process in patients with deficits in cognition and communication [25].

With the structured interview motor and self-care-related goals were again rated as highly relevant for rehabilitation by the majority of patients and paralleled the self-ratings of functional abilities and perceptions of acute problems from the patients’ perspective. But interestingly, psychological categories summarized as “psycho-social well-being” (handling stress, improve mood and cognition) were also rated as important rehabilitation goals by up to 50*%* of participants reflecting the high psychological burden of acute illness and hospitalization in our study sample. It reveals the importance attributed to psychological and cognitive functioning for quality of life and recovery by patients and strongly suggests an increased focus of GR concepts on psycho-social interventions and outcomes.

### Insight in acute functional deficits

The high percentage of deficits in functional and motor activities but also in activities related to self-care and psycho-social functioning reported by patients are in accordance with findings in other studies concerning the prevalence of impairments in older patients in GR in general [60]. However, we are not aware of comparable studies that have explored the insight in functional deficits as a consequence of an acute illness in geriatric rehabilitants with dementia. The majority of studies, exploring insight, focused on memory dysfunctions or on instrumental activities of daily living [28, 61, 62]. There has been only little research on the perception of everyday functional abilities with heterogeneous results. While some studies only found modest correlation between self-reports and actual performance in everyday activities as well as an overestimation of abilities in self-report increasing with severity of dementia [32, 33] others revealed moderate to high correlations between self-ratings and objectively assessed instrumental activities of daily living [34] comparable to the results of the present study. Our results have demonstrated sustained insight in acute functioning in a study sample of patients with mild to moderate dementia in the specific setting of post-acute inpatient GR. The significant correlations between self-ratings and objective clinical measures of walking abilities, lower muscle strength, mobility and activities of daily living suggest a sustained, coherent perception of functional health problems. Relations were highest between self-rated walking abilities and assessments of gait-performance, while relationship between subjective and objective measures for motor functioning, lower muscle strength and self-care were moderate. A plausible explanation for this finding could be the higher concept equivalence and similarity between the items of the questionnaire and the objective measurements by (I) matching very specific functional abilities and (II) therefore approximate isomorphism between questionnaire and activities that (III) could be monitored and judged in direct personal experience with the deficit in everyday situations. In addition, for the items “feeding” and “personal hygiene” the restricted ordinal data format of the clinical measure could have artificially lowered statistical power and correlation coefficients.

### Influence of cognitive status

Even though the degree of insight can vary inter-individually, the majority of studies concluded that awareness deficits increase with cognitive decline [28, 61]. Our results support these findings as we found less differentiated insight in actual problems in the sub-group of patients with lower cognitive status. However, the relationship between the severity of cognitive impairment and the respective awareness does not seem to be straightforward as there was considerable variation in both subgroups. While in the sub-group of patients with higher cognitive status correlations and trend tests reached statistical significance for all categories, significance was reached in some but not all tested categories in the subgroup with lower cognitive status. This finding is in line with results of a longitudinal study that assessed awareness in relation to memory, everyday activities, and socio-emotional functioning over 20 months and concluded that at least in the earlier stages of dementia awareness will not inevitably decrease as dementia progresses [63]. Patients who were unable to correctly perceive and judge their problems in a specific activity could demonstrate higher awareness in other contexts. The aforementioned differences again affected particularly those categories that showed the least correlations between subjective and objective measures for the whole sample, with low similarity between items of questionnaire and motor testing, thus offering only low structural correspondence, what may also indicate rather a methodological shortcoming than a distinctive difference of insight between groups.

Overall, our results contrast the generalized view that PwD are unable to perceive and evaluate their functional status [64–66], which may represent a staff related barrier for collaborative goal-setting leading to a systematic neglect of the patients´ perspective in favor of caregiver priorities.

### Limitations

Due to the lack of validated instruments, to support the identification and verbalization of potential rehabilitation goals in our cognitively impaired study population, we developed a tool with pre-selected goal categories for the structured goal assessment. We identified relevant categories of the comprehensive ICF core set for older adults in early post-acute rehabilitation facilities [49, 51] by a consensus process. Such a consensus process is based on long-term clinical expertise but may need further empirical research to ascertain that selected categories match the perceived problems and associated goals of patients with mild to moderate dementia in GR. The development of a specific ICF Core Set for PwD in geriatric post-acute rehabilitation should therefore be a next step to provide standards for multi-professional comprehensive patient assessment and should facilitate collaborative goal-setting with PwD in geriatric rehabilitation.

## Conclusion

Our results underline the sustained potential of patients with mild to moderate dementia to participate in goal-setting, especially when supported by a structured approach. Patients` health evaluations and most frequently reported goals reflect a prototypical spectrum of impairments, limitations and restrictions of PwD in GR. Patients demonstrated a sustained awareness for their functioning in activities related to key motor functions and daily living despite their cognitive impairment. However, clinicians should be aware that persons with lower cognitive status showed less differentiated insight in their acute problems.

The findings point to the necessity that clinicians have to reconcile their own and their patients’ valuations when they aim to come to a comprehensive understanding of meaningful rehabilitation goals as patients demonstrated a large spectrum of functional but also psychological rehabilitation needs which were not directly related to the patients` diagnosis at hand.

The present study has provided empirical data to support future lines of research focusing on the development of guidelines and practices for a dementia-specific assessment of goals to facilitate and promote collaborative goal-setting in PwD in rehabilitation practice.

## Data Availability

The datasets used and analyzed during the current study are available from the corresponding author on reasonable request.

## Abbreviations

1 RM: One repetition maximum leg press
ADL: Activity of Daily Living
BI: Barthel Index
GDS: Geriatric Depression Scale
GR: Geriatric Rehabilitation
HABA: Hierarchical Assessment of Balance and Mobility
ICF: International Classification of Functioning, Disability and Health
IQR: Interquartile range
MMSE: Mini Mental State Examinations
POMA: Tinetti’s Performance Oriented Motor Assessment
PwD: Patients with Dementia
SD: Standard Deviations
TuG: Timed up and go test
